# Toxin Production in Soybean (*Glycine max* L.) Plants with Charcoal Rot Disease and by *Macrophomina phaseolina,* the Fungus that Causes the Disease

**DOI:** 10.3390/toxins11110645

**Published:** 2019-11-06

**Authors:** Hamed K. Abbas, Nacer Bellaloui, Cesare Accinelli, James R. Smith, W. Thomas Shier

**Affiliations:** 1 Biological Control of Pests Research Unit, US Department of Agriculture-Agricultural Research Service, Stoneville, MS 38776, USA; 2 Crop Genetics Research Unit, US Department of Agriculture-Agricultural Research Service, Stoneville, MS 38776, USA; nacer.bellaloui@usda.gov (N.B.); rusty.smith@usda.gov (J.R.S.); 3 Department of Agricultural and Food Sciences, Alma Mater Studiorum–University of Bologna, 40127 Bologna, Italy; cesare.accinelli@unibo.it; 4 Department of Medicinal Chemistry, College of Pharmacy, University of Minnesota, Minneapolis, MN 55455, USA

**Keywords:** fungi, mycotoxins, phaseolinone, LC/MS, soybean, charcoal rot disease, root infection mechanism

## Abstract

Charcoal rot disease, caused by the fungus *Macrophomina phaseolina*, results in major economic losses in soybean production in southern USA. *M. phaseolina* has been proposed to use the toxin (-)-botryodiplodin in its root infection mechanism to create a necrotic zone in root tissue through which fungal hyphae can readily enter the plant. The majority (51.4%) of *M. phaseolina* isolates from plants with charcoal rot disease produced a wide range of (-)-botryodiplodin concentrations in a culture medium (0.14–6.11 µg/mL), 37.8% produced traces below the limit of quantification (0.01 µg/mL), and 10.8% produced no detectable (-)-botryodiplodin. Some culture media with traces or no (-)-botryodiplodin were nevertheless strongly phytotoxic in soybean leaf disc cultures, consistent with the production of another unidentified toxin(s). Widely ranging (-)-botryodiplodin levels (traces to 3.14 µg/g) were also observed in the roots, but not in the aerial parts, of soybean plants naturally infected with charcoal rot disease. This is the first report of (-)-botryodiplodin in plant tissues naturally infected with charcoal rot disease. No phaseolinone was detected in *M. phaseolina* culture media or naturally infected soybean tissues. These results are consistent with (-)-botryodiplodin playing a role in the pathology of some, but not all, *M. phaseolina* isolates from soybeans with charcoal rot disease in southern USA.

## 1. Introduction

The fungus *Macrophomina phaseolina* (Tassi) Goidanich [1], also known by the teleomorph *Sclerotium bataticola* Taub. [2], is the cause of charcoal rot disease, and other named diseases, in soybeans and about 500 other crop and ornamental species in the United States and internationally [3,4,5]. Charcoal rot disease, also known as summer wilt, dry weather wilt, or black root disease, results in crop yield loss and seed quality deterioration in soybeans and other crops [6,7,8,9,10,11]. Charcoal rot disease is more prevalent in heat- and drought-stressed conditions [12,13]. *M. phaseolina* can spread to adjacent plants with interdigitating roots through the soil, infecting the roots and spreading throughout the infected plant through the vascular system [14,15]. *M. phaseolina* forms black spore-like mycelial structures called microsclerotia, which allow the fungus to survive over winter. These microsclerotia are the grey and black dots in the stems and roots of soybean plants that give charcoal rot disease its name [16]. Common agricultural practices such as managing planting dates, fungicide applications, and biological control have been ineffective in controlling this disease [17,18,19,20,21,22,23]. Despite extensive efforts to control charcoal rot disease by developing resistant soybean genotypes [24,25,26], currently available genotypes are still not sufficiently resistant to prevent the disease in the field, although moderately resistant genotypes have been shown to have lower levels of *M. phaseolina* in plant tissues [27,28,29].

The mechanism used by *M. phaseolina* to infect plants with charcoal rot disease is not yet understood, in part because of the diversity in *M. phaseolina* isolates [30,31,32,33]. *M. phaseolina* has been reported to produce toxins, including (-)-botryodiplodin and phaseolinone [34,35,36,37]. It has been proposed that a toxin may play a role in an early step of the mechanism used by *M. phaseolina* to infect susceptible plants through the roots from the soil reservoir, where the fungus normally lives, particularly over the winter [7,36].

The objective of the present study is to investigate the involvement of toxins, particularly (-)-botryodiplodin, in the charcoal rot disease of soybeans. Soybeans are selected as the subject for these studies because charcoal rot disease causes major economic losses for soybean production in the midsouthern USA (Mississippi, Arkansas, and Louisiana) [10,11,38,39,40]. The role(s) of toxins in root infection is investigated in these studies by assessing the production of (-)-botryodiplodin, phaseolinone, and other toxins in cell-free culture filtrates of charcoal rot disease-causing *M. phaseolina* isolates and in roots and other tissues from soybean plants naturally infected with charcoal rot disease in the field. Studies on the culture filtrates of charcoal rot disease-causing *M. phaseolina* isolates resulted in the discovery that some, but not all, isolates produce (-)-botryodiplodin, but not phaseolinone, and some isolates that do not produce (-)-botryodiplodin do produce another as yet unknown toxin(s). Studies on toxins present in soybean plant tissues provided the first demonstration of a mycotoxin known to be produced by *M. phaseolina* in soybean plant tissues naturally infected with charcoal rot disease, specifically (-)-botryodiplodin, but not phaseolinone.

## 2. Results and Discussion

### 2.1. Toxin Production in Culture by M. Phaseolina Isolates from Plants with Charcoal Rot Disease

Toxin production in culture by *M. phaseolina* isolates from many USA sites and numerous types of plant sources were examined as the toxicity of cell-free culture medium filtrates in soybean leaf disc cultures from two soybean genotypes, DS97-84-1 and DT97-4290 (Table 1). Toxicity assessments with the two genotypes exhibited a similar rank order with no substantive difference between the two, whether assessed at 50% strength or at full strength. The same cell-free culture filtrates from *M. phaseolina* isolates were also assayed by LC/MS for levels of (-)-botryodiplodin, the toxin previously [35] found associated with culture filtrates of a *M. phaseolina* isolate from a soybean plant in Mississippi with charcoal rot disease. Observed concentrations of (-)-botryodiplodin ranged from not detectable to 6.11 µg/mL (Table 1). The majority of isolates (51.4% of isolates studied) produced quantifiable levels of (-)-botryodiplodin in culture filtrates, while 37.8% of isolates studied produced trace levels (i.e., above the limit of detection (1 × 10^−5^ ng/µL), but less than the limit of quantitation (1 × 10^−2^ ng/µL), and 10.8% of isolates studied produced no detectable level of (-)-botryodiplodin in culture filtrates.

Whether *M. phaseolina* isolates were from trees, soybeans, melons, or other plant sources, cell-free culture filtrates were toxic in soybean leaf disc cultures, and toxicity levels varied from not detectable to very toxic (Table 1). Culture filtrates from *M. phaseolina* isolates that contained high levels of (-)-botryodiplodin (>1 µg/mL) were all very toxic in soybean leaf disc cultures, resulting in maximal or near maximal toxicity with both DT97-4290 and DS97-84-1 soybean leaf discs at 100% and 50% strength. Culture filtrates that contained intermediate levels of (-)-botryodiplodin (0.2–1.0 µg/mL) were moderately toxic in soybean leaf disc cultures. However, some other *M. phaseolina* isolate culture filtrates that contained only trace levels or even no detectable (-)-botryodiplodin were highly toxic in soybean leaf disc cultures. This observation is consistent with some disease-inducing isolates of *M. phaseolina* producing one or more toxins other than (-)-botryodiplodin. This is the first report of results supporting the hypothesis that different isolates of *M. phaseolina* may use different toxins to facilitate root infection in soybeans. Further studies are needed to determine if any of those isolates use the other toxin(s) to facilitate root infection by a mechanism analogous to the one by which (-)-botryodiplodin might facilitate root infection. Some culture filtrates from the disease-inducing isolates of *M. phaseolina* contained very little toxicity in soybean leaf disc cultures, despite the isolate being able to cause charcoal rot disease. Explanations for this observation include the possible presence of a toxin-production regulatory mechanism that suppresses toxin production by those isolates under the culture conditions used in this study, or the possibility that charcoal rot disease in soybeans may be caused by a seed-borne *M. phaseolina* endophyte that would not need a root infection mechanism or any toxins associated with it [14]. The *M. phaseolina* isolate from which phaseolinone was originally isolated [34,37] was a seed-borne endophyte.

Also included in Table 1 is an assessment of the color of week-old cultures of *M. phaseolina*. Dunlap and Bruton [41] reported that a *M. phaseolina* isolate formed pigment in an infected muskmelon (*Cucumis melo*) and in liquid culture media containing glycine and some other amino acids. Some *M. phaseolina* isolates that cause charcoal rot disease in soybeans have been observed to form large numbers of black microschlerotia under the same culture conditions that induce the production of (-)-botryodiplodin [42]. In the data in Table 1, the rank order of pigment production, as assessed qualitatively according to the color density scale used, differed substantially from the rank order of toxicity in cell-free culture filtrates as assessed in soybean leaf disc cultures at either full strength or 50% dilution and from the relative amount of (-)-botryodiplodin present as measured by LC/MS. Thus, pigment production as assessed in this study appeared to be unrelated to toxin production, consistent with the previously identified correlations not being a general phenomenon when larger numbers of *M. phaseolina* isolates are examined. 

### 2.2. Analysis of Toxin Levels in Tissue Samples from Soybean Plants Naturally Infected with Charcoal Rot Disease

If the hyphae of a *M. phaseolina* strain that causes charcoal rot disease use a toxin(s) to create a necrotic area in the root and thereby facilitate entry into soybean plant roots from a soil reservoir, those hyphae are expected to produce a toxin(s) at least from the time the fungus detects the root in the soil until the fungal hyphae inside the plant have detected that a stable infection has been established there. Fungi that spread from plant to plant through interdigitating roots, as *M. phaseolina* does in the charcoal rot disease of soybeans, may also secrete a toxin(s) inside the roots of fully infected plants in order to create a necrotic area within the root from which hyphae may exit the plant to spread to adjacent plants. Thus, soybean plants exhibiting the symptoms of charcoal rot disease may contain a chemically and metabolically stable toxin in tissues at a level detectable by standard analytical methods such as LC/MS. If the *M. phaseolina* strain causing charcoal rot disease in a soybean plant is a constitutive (continuous) producer of the toxin, comparable levels of the toxin may be expected in all the affected tissues of diseased plants. Therefore, naturally infected soybean plants exhibiting symptoms of charcoal rot disease were collected from different infected areas in commercial soybean production fields in Mississippi in the 2004 growing season. Control soybean plants not exhibiting symptoms of charcoal rot disease were also collected. Samples of roots, leaves, stem pulp, branches, twigs, and seeds were individually extracted and analyzed by LC/MS for levels of (-)-botryodiplodin, phaseolinone, phomenone, and gigantenone (Table 2). Only (-)-botryodiplodin was detected and only in the roots of soybean plants exhibiting symptoms of charcoal rot disease, not in other tissues of diseased plants and not in the roots or any other tissues of control soybean plants not exhibiting symptoms of charcoal rot disease. This is the first report of a toxin being found in infected plant tissues associated with charcoal rot disease in soybeans. This observation would be expected if *M. phaseolina* used (-)-botryodiplodin in its mechanism for (i) initial root infection and (ii) to exit heavily infected plants in order to spread to and infect adjacent plants. However, additional studies are needed to establish a role for (-)-botryodiplodin in either the initial root infection or the root exit mechanism. No phaseolinone, phomenone, or gigantenone was found in any tissue of soybean plants with charcoal rot disease in this study. As shown in Table 3, these observations are confirmed by a similar study conducted in 2007, in which root tissue was collected from naturally infected soybean plants from commercial production fields in Mississippi and Kentucky, USA, and analyzed by LC/MS for levels of (-)-botryodiplodin, phaseolinone, phomenone, and gigantenone. As observed in the first study (Table 2), only (-)-botryodiplodin was detected in diseased roots, not phaseolinone, phomenone, or gigantenone. Again, (-)-botryodiplodin levels varied from traces to 3.14 µg/g, that is, greater than a 1000-fold concentration range. The wide range of (-)-botryodiplodin levels in charcoal rot-diseased soybean roots (Table 2 and Table 3) paralleled the wide range of (-)-botryodiplodin production levels in cell-free culture filtrates of *M. phaseolina* isolates from plants with charcoal rot disease. In both experimental systems, there were a substantial number of cases in which (-)-botryodiplodin production was too low for it to be a toxin that could play a role in the pathology caused by those *M. phaseolina* strains, whether by facilitating root infection or any other mechanism.

## 3. Conclusions

A wide range of (-)-botryodiplodin levels were observed in both cell-free culture medium filtrates from *M. phaseolina* isolates from plants with charcoal rot disease and in the roots, but not in the aerial parts, of soybean plants naturally infected with charcoal rot disease. Cell-free culture medium filtrates from some *M. phaseolina* isolates from plants with charcoal rot disease were strongly phytotoxic, despite containing only traces or no (-)-botryodiplodin. No phaseolinone was detected in either cell-free culture medium filtrates from *M. phaseolina* isolates or in tissues from soybean plants naturally infected with charcoal rot disease. The results of this study are consistent with some, but not all, isolates of *M. phaseolina* associated with charcoal rot disease in soybean-producing (-)-botryodiplodin. Some isolates of *M. phaseolina* cultured from soybean plants with charcoal rot disease produce no detectable (-)-botryodiplodin in culture, but do produce other unknown toxins. Further research is needed to determine what role, if any, (-)-botryodiplodin and other toxins produced by *M. phaseolina* isolates play in the root infection mechanism of the charcoal rot disease of soybeans. 

## 4. Materials and Methods

### 4.1. Soybean Plant and Greenhouse Conditions

The soybean genotype DT97-4290 [28] was selected as an example of a genotype that is moderately resistant to charcoal rot disease, and the soybean genotype DS97-84-1 [43] was selected as an example of a genotype that is susceptible to charcoal rot disease. Plants were germinated in trays with vermiculite, and the seedlings of each genotype were transplanted into six soil-filled 9.45 L pots, each containing four plants of the same genotype. During the growth period, the soil water potential of the plants was maintained at approximate field conditions, 15 to −20 kPa. Six pots, each containing four plants, were used for each genotype. The greenhouse temperature was set to 34 °C for the day cycle and 28 °C for the night cycle. Light intensity ranged from that of sunny to cloudy days. Plants were harvested during the vegetative stage.

### 4.2. M. phaseolina Culture Sources

The collection locations and plant hosts of the 37 cultures of *M. phaseolina* used in the study are presented in Table 1. Some *M. phaseolina* cultures were isolated from infected plant tissues in the Abbas laboratory in 2013 using the method of Mengistu et al. [4,25], while other cultures were provided by colleagues from their collections, particularly G.L. Sciumbato, Mississippi State University.

### 4.3. Preparation of Cell-Free Culture Extracts

Potato dextrose broth (PDB) was prepared by boiling 200 g of peeled potatoes, straining them through a cheesecloth, and adding 20 g of dextrose per liter of water. PDB (150 mL) was placed in 500 mL Erlenmeyer flasks, covered with cotton plugs, autoclaved for 15 min, and allowed to cool to room temperature. Upon cooling, each flask was inoculated with three to four plugs of *M. phaseolina* isolate and placed on an Innova 40 Benchtop Incubator Shaker (New Brunswick Scientific Co., Inc., Edison, NY, USA) for seven days at 128 rpm, 28 °C. The color change of each culture after one week of incubation was observed and recorded according to the following color density scale: whitish < light yellow < light tan < light grey < tan < beige or amber < dark tan < dark brown or dark grey < black. 

After seven days of incubation, the culture medium was passed through Whatman No.1 filter paper into a plastic beaker. The filtrate was then filtered through an 0.45 µm membrane filter in a disposable filter unit (Nalgene Company, Rochester, NY, USA, Size 250 mL cellulose nitrate CN Filter Unit) using a laboratory vacuum to achieve a cell-free filtrate that was stored at −20 °C until used.

### 4.4. Toxicity of Cell-Free Filtrates of M. phaseolina Culture Media in Soybean Leaf Disc Cultures

The toxicity of *M. phaseolina* culture filtrates was assessed by rating the appearance of soybean leaf discs from two genotypes (DT97-4290, which is moderately resistant to charcoal rot, and DS97-84-1, which is susceptible) after four to five days in half (50%) and full strength (100%) cell-free culture filtrates, *M. phaseolina* isolates were grown on potato dextrose agar (PDA) for seven days at 28 °C. True mature leaves with no signs of damage were harvested from 3- to 4-week-old soybean plants, and 4 mm discs were cut from the leaves using a sterile cork borer (No.4). Three leaf discs were placed in each well of sterile 24-well tissue culture trays with low evaporative lids (Becton Dickinson and Company, Franklin Lakes, NJ, USA) containing 1.5 mL of culture filtrate in triplicate at two concentrations (50% and 100%). The trays were then incubated in a growth chamber at 25 °C under continuous light for 96 h. The discs were observed for signs of toxic effects after 24, 48, 72, and 96 h. Toxicity was assessed qualitatively according to the following symptom rating scale: healthy tissue < a little browning around the edges of the leaf disc, + < moderate browning around the edges of the leaf disc, ++ < browning of the whole leaf disc, +++ < browning of the leaf disc with some photobleaching, ++++ < photobleaching of the whole leaf disc, +++++.

### 4.5. Toxin Standards for LC/MS Analyses

**Figure 1:**
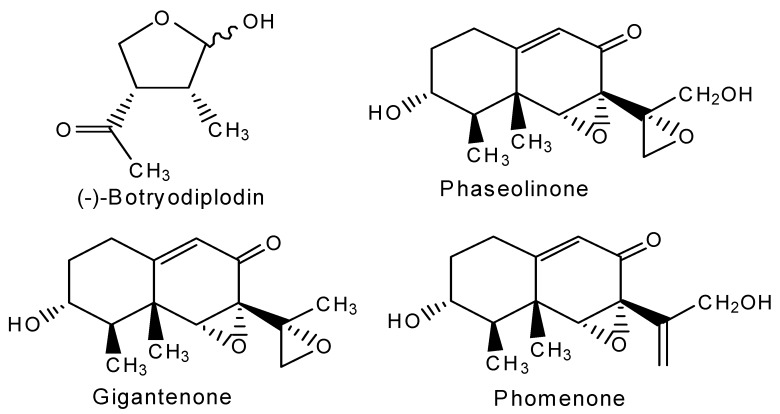
Chemical structures of the toxins measured by LC/MS in *M. phaseolina* culture media and soybean root tissues.

**Figure 2:**
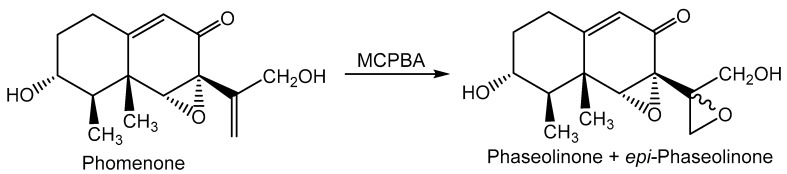
The chemical reaction used in the semi-synthesis of the LC/MS standard phaseolinone from the natural toxin phomenone. MCPBA = meta-chloroperoxybenzoic acid.

The structures of toxins measured in this study are presented in Figure 1. (±)-Botryodiplodin was synthesized, as described in the accompanying manuscript [44], as a white powder with purity over 98%. A stock solution of (±)-botryodiplodin (1000 ng/µL) was prepared in chloroform. Working standards were prepared in the concentration range 1.0 × 10^−5^ ng/µL to 40 ng/µL in ethyl acetate. Gigantenone and phomenone were gifts from Gary A. Strobel, Montana State University, Bozeman, MT. Phaseolinone was synthesized (Figure 2) from a sample of the phomenone (6.5 mg, 0.0246 mmole) dissolved in 1 mL chloroform and mixed with a 1.2 molar excess of m-chloroperoxybenzoic acid (Acros Organics, 0.029 mmole, 7.15 mg of 70% pure material) and pyridine (4.7 μL, 4.6 mg, 0.058 mmole) dissolved in 200 μL chloroform. The mixture was incubated for 1 h at −10 °C with stirring and then allowed to warm to room temperature overnight. The reaction mixture was diluted with ether, extracted twice with water, once with 1N HCl to remove pyridine, twice with saturated sodium bicarbonate-brine solution to remove product m-chlorobenzoic acid and unreacted m-chloroperoxybenzoic acid, dried over anhydrous sodium sulfate, and evaporated in vacuo. The product (7.5 mg) gave a single peak at *m/e* 281 (phaseolinone + H^+^) in LC/MS analysis under the conditions described below, with no detectable phomenone starting material at *m/e* 265. A single peak was observed in LC/MS for the phaseolinone preparation, even though the reaction conditions would be expected to produce a mixture of phaseolinone and epi-phaseolinone, presumably because the two forms were not resolved under the liquid chromatography conditions used.

### 4.6. Preparation of Plant Tissue and M. phaseolina Culture Medium Extracts for LC/MS Analyses

Soybean root and other tissue samples were cleaned of adherent earth, dried in an oven at 45 °C for two to three days, and ground to the consistency of flour using a Stain Laboratory Mill Grinder, Model M-2 (Fred Stein Laboratories, INC., Atchison, Kansas, USA). Ethyl acetate (10 g) was added to 50 g of each sample, shaken for 1 h, filtered through filter paper (Whatman No.1), and transferred to vials for analysis by LC/MS as described below. *M. phaseolina* culture medium cell-free filtrate samples were extracted with ethyl acetate in a 1:1, *v*:*v* ratio on a vortex mixer for 1 min and allowed to separate into two distinct layers. The ethyl acetate layer was transferred to vials for analysis by LC/MS.

### 4.7. LC/MS Analysis

LC/MS analyses of toxin samples obtained prior to 2007 were conducted on a Thermo Finnigan LCQ Advantage instrument coupled to a Thermo Finnigan Surveyor MS and a Thermo Finnigan Surveyor MS Pump (Thermo Electron Corporation, West Palm Beach, FL, USA). After 2007, a more advanced and upgraded LTQ XL Ion Trap Mass Spectrometer, Finnigan Surveyor Autosampler, and Finnigan Surveyor MS Pump (Thermo Scientific, West Palm Beach, FL, USA) were used. Analyses were carried out in positive scan mode at ambient temperature using a Waters Nova-Pak C18 column, a 10 µL partial loop injection, and mobile phases (A) 1% acetic acid in methanol, (B) water, and (C) methanol at a flow rate of 500 µL/min. The analysis occurred over 25 min using a gradient of 20% A and 80% B for 12 min, then 20% A, 5% B, and 75% C for 3 min, and then back to 20% A and 80% B for the duration of the 25 min. The analysis utilized the following scan events of a full scan from *m/e* 100 to 300. The confirmation of (-)-botryodiplodin used three masses: *m/e* 127, 145, and 109. The limit of detection (LOD) was 1 × 10^−5^ µg/mL and the limit of quantitation (LOQ) was 0.01 µg/mL. The LOQ was based on the regression of the standards used for analysis. The full scan run of phomenone, gigantenone, and phaseolinone was from *m/e* 100 to 500, and their confirmations were identified by using *m/e* 265, 265, and 281, respectively.

### 4.8. Statistical Analysis

The analysis of variance was performed on toxin concentration data using the PROC GLM procedure in SAS Version 9.22 (Cary, NC, USA, 2010). Means were separated by Fisher’s Least Significant Difference test with *p* ≤ 0.05 level of significance.

## Figures and Tables

**Table 1:** Toxicity, color, and (-)-botryodiplodin production in cell-free culture medium filtrates from *Macrophomina phaseolina* isolates from plants exhibiting charcoal rot disease properties in various states of the USA.

			Toxicity ^a^ in Leaf Disc Cultures of Two Soybean Genotypes					
			DS97-84-1		DT97-4290			
Isolate	Collection Site	Plant Host	50% Strength	100% Strength	50% Strength	100% Strength	Color ^b^ in One-Week Cultures	(-)-Botryodiplodin ^c^ Concentration (µg/mL)
*Mp*001A	MS	Soybean	++	++	+	+	beige	Trace
*Mp*001B	MS	Soybean	+	+	+	+	l tan	0.19
*Mp*004	MS	Soybean	++++	++++	+++	++++	d tan	0.18
*Mp*006A	MS	Soybean	++	++	+	++	l yellow	Trace
*Mp*006B	MS	Soybean	++	+++	+	+	l tan	0.14
*Mp*007	MS	Soybean	++	+++	++	+++	beige	0.29
*Mp*008A	MS	Soybean	+	+	+	+	l yellow	0.15
*Mp*008B	MS	Soybean	++	+++	++	+++	beige	0.14
*Mp*009A	MS	Soybean	++	++	+	+	l tan	Trace
*Mp*010B	MS	Soybean	++	++	+	+	l tan	Trace
*Mp*011A	MS	Soybean	++	++	+++	+++	l tan	Trace
*Mp*144	KY	Soybean	+++	+++	++	+++	beige	0.18
*Mp*146	KY	Soybean	+++	+++	++	++	beige	0.2
*Mp*176	AR	Soybean	+	+	+	+	l yellow	0.08
*Mp*178	AR	Soybean	+++++	+++++	+++++	+++++	beige	1.64
*Mp*203	LA	Soybean	+++	+++	+++	+++	tan	Trace
*Mp*204	LA	Soybean	++	++	+	+	l tan	Trace
*Mp*214	SD	Soybean	+++	++++	+++	+++	l yellow	Trace
*Mp*220	TN	Soybean	++	++	+	+	l tan	0.17
*Mp*223	TX	Soybean	+++	+++	++++	++++	tan	0
*Mp*242	ND	Soybean	+	+	+	+	l tan	0.16
*Mp*272	MN	Soybean	++++	++++	++++	++++	l tan	Trace
*Mp*279	OK	Soybean	+++++	+++++	++++	+++++	d grey	4.03
*Mp*302	KS	Soybean	+++	+++	++	++	l tan	0.98
*Mp*305	KS	Soybean	++++	++++	+++	+++	l tan	Trace
*Mp*251	NE	Dry bean	++++	+++++	++++	+++++	tan	4.5
*Mp*228	NC	Fraser fir	+	+	+	+	l yellow	0
*Mp*264	MI	Fir	+++++	+++++	+++++	+++++	tan	6.11
*Mp*275	MN	Redwood	+++++	+++++	++++	+++++	tan	Trace
*Mp*183	FL	Strawberry	+++	++++	+++	++++	tan	0.74
*Mp*315	AZ	Watermelon	++++	+++++	++++	+++++	beige	Trace
*Mp*249	GA	Unknown	+++++	+++++	++++	++++	tan	2.04
*Mp*216	Unknown	Unknown	++++	++++	+++	+++	l grey	0.31
*Mp*234	Unknown	Unknown	++++	++++	++++	++++	tan	0
*Mp*235	Unknown	Unknown	+	+	+	+	l tan	Trace
*Mp*238	Unknown	Unknown	+	+	+	+	l tan	0
*Mp*239	Unknown	Unknown	+	+	+	+	l tan	Trace

Abbreviations: *Macrophomina phaseolina, Mp*; Mississippi, MS; Kentucky, KY; Arkansas, AR; Louisiana, LA; South Dakota, SD; Tennessee, TN; Texas, TX; North Dakota, ND; Minnesota, MN; Oklahoma, OK; Kansas, KS; Nebraska, NE; North Carolina, NC; Michigan, MI; Florida, FL; Arizona, AZ; Georgia, GA; light, l; dark, d. ^a^ Toxicity score measured in soybean leaf disc cultures of two soybean genotypes: (i) DT97-4290, which is moderately resistant to charcoal rot disease and (ii) DS97-84-1, which is susceptible to charcoal rot disease. Toxicity was assessed qualitatively according to the following symptom rating scale: healthy tissue < a little browning around the edges of the leaf disc, + < moderate browning around the edges of the leaf disc, ++ < browning of the whole leaf disc, +++ < browning of the leaf disc with some photobleaching, ++++ < photobleaching of the whole leaf disc, +++++. ^b^ Color density was assessed qualitatively according to the following color density scale: whitish < light yellow < light tan < light grey < tan < beige or amber < dark tan < dark brown or dark grey < black. ^c^ (-)-Botryodiplodin concentrations in culture medium filtrates were measured quantitatively by LC/MS.

**Table 2:** Mycotoxin levels in root and other tissues of soybean plants collected from soybean fields in Mississippi in 2004.

Sample Name	Charcoal Rot Disease	Soybean Tissue Type	(-)-Botryodiplodin (µg/g) ^a^	Phomenone (µg/g) ^a^	Gigantenone (µg/g) ^a^	Phaseolinone (µg/g) ^a^
RTS 999 302	Yes	Roots	0.786	0	0	0
USG 7582 306	Yes	Roots	0.13	0	0	0
DK B58-51 326	Yes	Roots	0.23	0	0	0
AG 5903 324	Yes	Roots	0.046	0	0	0
AG 5701 329	Yes	Roots	0.139	0	0	0
PGX 5703 313	Yes	Roots	0.334	0	0	0
PGY 5822 319	Yes	Roots	0.332	0	0	0
ESXVT-46 328	Yes	Roots	0.134	0	0	0
P 95 B96 301	Yes	Roots	0.006	0	0	0
P GX 5714 311	Yes	Roots	0.141	0	0	0
Garst 5812 331	Yes	Roots	0.209	0	0	0
DK 5767 32	Yes	Roots	0.061	0	0	0
All samples tested	Yes	Seeds	0	0	0	0
All samples tested	Yes	Pulp	0	0	0	0
All samples tested	Yes	Branches	0	0	0	0
All samples tested	Yes	Twigs	0	0	0	0
All samples tested	Yes	Leaves	0	0	0	0
Undiseased control ^b^	No	Roots	0	0	0	0

^a^ Identification and quantification of toxins in samples by LC/MS were based on one standard due to the limited amount of standards available. ^b^ Soybean plants of the Saline cultivar with no detectable sign of charcoal rot disease were collected from commercial fields in Mississippi. Samples of the same six tissues were taken, pooled, and extracted in the same way as tissues from diseased plants and the extracts were assayed by LC/MS in the same manner. Extracts of all undiseased soybean tissues, including roots, contained no detectable (-)-botryodiplodin or other toxin.

**Table 3:** Toxins in the roots of soybean plants exhibiting charcoal rot disease properties collected from commercial soybean fields in Kentucky and Mississippi in 2007.

Field and Location *	(-)-Botryodiplodin (µg/g)	Phomenone (µg/g)	Gigantenone (µg/g)	Phaseolinone (µg/g)
1 KY	0.870	0	0	0
417 MS	trace	0	0	0
1 KY	0.567	0	0	0
314 MS	3.139	0	0	0
3 KY	0.114	0	0	0
4 KY	0.115	0	0	0
2 KY	0.938	0	0	0
209 MS	0.757	0	0	0
4 KY	0.946	0	0	0
312 MS	0.703	0	0	0
210 P12 MS	trace	0	0	0

* Soybean plants exhibiting symptoms of charcoal rot disease were collected in the indicated commercial field numbers in the indicated states, brought to the laboratory, tissues harvested and stored at −20 °C until assayed. Soybean root samples had symptoms of charcoal rot and were run by LC/MS. Determination and quantification of these mycotoxins was by LC/MS based on one standard because a limited amount of these standards were available.
